# Codelivery of resveratrol melatonin utilizing pH responsive sericin based nanocarriers inhibits the proliferation of breast cancer cell line at the different pH

**DOI:** 10.1038/s41598-023-37668-y

**Published:** 2023-07-08

**Authors:** Faranak Aghaz, Zahra Asadi, Soraya Sajadimajd, Khosrow Kashfi, Elham Arkan, Zohreh Rahimi

**Affiliations:** 1 Nano Drug Delivery Research Center, Health Technology Institute, Kermanshah University of Medical Sciences, Kermanshah, Iran; 2 Students Research Committee, Kermanshah University of Medical Sciences, Kermanshah, Iran; 3 Department of Clinical Biochemistry, Medical School, Kermanshah University of Medical Sciences, Kermanshah, Iran; 4 Department of Biology, Faculty of Science, Razi University, Kermanshah, Iran; 5 Department of Molecular, Cellular and Biomedical Sciences, Sophie Davis School of Biomedical Education, City University of New York School of Medicine, New York, USA; 6 Medical Biology Research Center, Health Technology Institute, Kermanshah University of Medical Sciences, Kermanshah, Iran

**Keywords:** Cancer, Cell biology

## Abstract

Protein-based nanocarriers have demonstrated good potential for cancer drug delivery. Silk sericin nano-particle is arguably one of the best in this field. In this study, we developed a surface charge reversal sericin-based nanocarrier to co-deliver resveratrol and melatonin (MR-SNC) to MCF-7 breast cancer cells as combination therapy. MR-SNC was fabricated with various sericin concentrations via flash-nanoprecipitation as a simple and reproducible method without complicated equipment. The nanoparticles were subsequently characterized for their size, charge, morphology and shape by dynamic light scattering (DLS) and scanning electron microscope (SEM). Nanocarriers chemical and conformational analysis were done by fourier transform infrared spectroscopy (FT-IR) and circular dichroism (CD) respectively. In vitro drug release was determined at different pH values (7.45, 6.5 and 6). The cellular uptake and cytotoxicity were studies using breast cancer MCF-7 cells. MR-SNC fabricated with the lowest sericin concentration (0.1%), showed a desirable 127 nm size, with a net negative charge at physiological pH. Sericin structure was preserved entirely in the form of nano-particles. Among the three pH values we applied, the maximum in vitro drug release was at pH 6, 6.5, and 7.4, respectively. This pH dependency showed the charge reversal property of our smart nanocarrier via changing the surface charge from negative to positive in mildly acidic pH, destructing the electrostatic interactions between sericin surface amino acids. Cell viability studies demonstrated the significant toxicity of MR-SNC in MCF-7 cells at all pH values after 48 h, suggesting a synergistic effect of combination therapy with the two antioxidants. The efficient cellular uptake of MR-SNC, DNA fragmentation and chromatin condensation was found at pH 6. Nutshell, our result indicated proficient release of the entrapped drug combination from MR-SNC in an acidic environment leading to cell apoptosis. This work introduces a smart pH-responsive nano-platform for anti-breast cancer drug delivery.

## Introduction

Breast cancer (BC) is the most prevalent malignancy and the leading cause of cancer death among women globally, accounting for 14.7% of all cancer-related deaths^1^. Notwithstanding significant advances in understanding and treating BC over the past decade, the incidence and mortality rate related to BC continue to increase at an alarming rate accounting for approximately 2.26 million deaths by 2020^2,3^. The International Agency for Research on Cancer (IARC) Global Cancer Observatory (GLOBOCAN) predicts 27.5 million new cancer cases per year by 2040, a 60% increase compared to the current statistics^4^. Current treatment modalities include any or a combination of surgery, radiation, immunotherapy, gene therapy, photodynamic treatment, and chemotherapy. Although these procedures can alleviate breast cancer, they have numerous disadvantages, including non-specificity, high toxicity, and multidrug resistance^5^. Thus, there is a need for alternative treatments for BC. Antioxidants have been widely used as chemo preventive agents to counteract BC progression. They can also be used as adjuvants in treating BC. Combining multiple antioxidants is one of most effective strategies in cancer management^6^.

**Figure 1:**
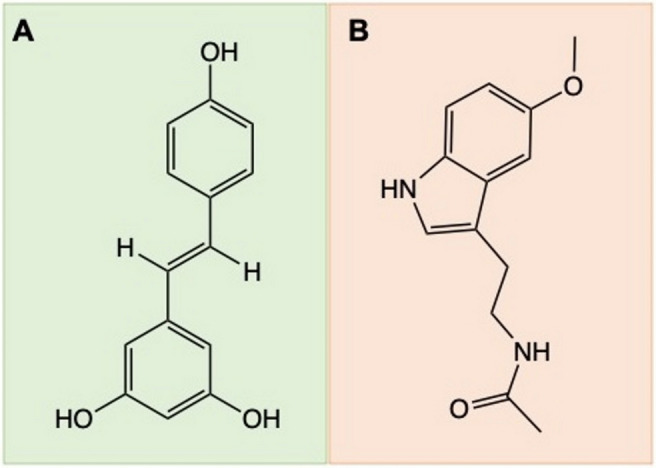
Structures of resveratrol (**A**) and Melatonin (**B**).

Resveratrol (3,4,5-*trans*-trihydroxystilbene, Fig. 1A) is a natural polyphenol which is naturally derived from plants especially grapes^7^. Polyphenols are a large category of natural phenol group with therapeutic and preventive effects on neurodegenerative disorders, cardiovascular disease and cancer, due to the beneficial properties of phenolic and polyphenolic compounds including anti-inflammatory, antioxidant and anticancer activities^8,9^.

Resveratrol cancer preventive properties were first reported in 1977^7^ followed by many in vivo and in vitro studies underscoring its role in modulating multiple signaling pathways^10^. It has antioxidant properties through scavenging free radicals, attenuates reactive oxygen species (ROS) generation, and inhibits cancer initiation and cancer development^11^.

Melatonin an indoleamine (N-[2-(5-methoxy-1H-indol-3-yl)ethyl]acetamide or N-Acetyl-5-methoxytryptamine, Fig. 1B) is a pineal gland hormone regulating the sleep–wake cycle, gonadal activity, redox homeostasis, and immune functions^12^. It also has anti-tumor properties in different types of cancer, including breast, colorectal, pancreatic, endometrial, kidney, skin, and others^13–19^. Of note, melatonin lowers estrogen receptor expression, inhibits DNA replication, prevents metastasis, regulates angiogenesis, and apoptosis^20,21^.

Recent advances in nanomedicine have ushered in a new era in cancer treatment by targeting cancer cells without affecting normal cells, thus potentially overcoming multidrug resistance. However, cancer heterogeneity and the tumor microenvironment complexity may attenuate the anti-cancer properties of nanocarriers^22^. Recent data strongly suggest that multifunctional nanocarriers offer enhanced efficacy, with limited side effects by modulating the local internal milieu such as redox conditions, temperature, pH, and actions of specific enzymes; or by external stimuli, such as ultrasound, light, and magnetic field^23^.

For two reasons, protein-based-charge-reversal nanocarrier development is one of the most promising strategies in this area. The most important reason is their pH-triggered charge-reversal property. Thus, they can absorb the negative-to-positive charge transition in response to changes in ambient pH. In fact, after accumulation in the tumor's acidic microenvironment, they obtain a positive charge that facilitates their cellular uptake^24,25^. The other reason is their protein-based nanocarrier drug delivery systems (P-NDDS) properties. P-NDDSs can have a significant role in BC treatment due to their beneficial characteristics^26,27^. For example, they are generally recognized as safe (GRAS)^28^, meaning there isn't any risk in using these carriers. They are also biodegradable—so over time, they break down inside the body. Another advantage of P-NDDSs that makes them attractive is their lack of immunostimulatory activity^27^. Thus, they don't trigger an immune response when administered^28^.

Silk sericin is one of the best proteins for developing a protein-based pH-triggered charge-reversal nanocarrier because of the pH-responsive nature of its building blocks. It is a 20–400 kDa protein extracted from dried silk cocoons, constituting about 25% of the total cocoon weight. This globular protein has 18 polar amino acids with amino, hydroxyl, and carboxyl groups^29^. They give it a hydrophilic property and a high antioxidant activity via scavenging free radicals. In addition, sericin is a stable, non-toxic, biodegradable, and biocompatible protein that interacts with other molecules easily. Besides its high antioxidative properties, it has a high potential to form a soluble and stable nanoparticle to wrap various compounds. The wrapping provides a long blood circulation time for entrapped compounds, prevents their decomposition, and releases them at specific targets^29–31^.

Previously published articles have shown that the nanoprecipitation method, also known as flash-nanoprecipitation, is a facile, mild, and low-energy input technique for nanocarrier preparation. It is also one of the most useful and safest strategies for encapsulating active molecules at sub-micron and nanoscale levels^32,33^. In fact, nanoprecipitation is a modest "bottom-up" approach that depends on the supersaturation of a hydrophobic solute upon adding water that has lately emerged as a general and flexible technique to produce nanoencapsulation with precise control of its physico-chemical characteristics^33,34^.

Therefore, this study focused on developing a novel pH-triggered charge reversal–protein-based nanocarrier using silk sericin employing the nanoprecipitation method. This nanocarrier was used to co-deliver resveratrol and melatonin as an antioxidant combination therapy to MCF-7 breast cancer cells. After the physico-chemical investigations of the fabricated nanoparticles, MR-SNC in vitro drug release behavior was studied at three pH values (6, 6.5, and 7.4). In addition, the MR-SNC potential to decrease the MCF-7 cells’ viability was investigated at different pHs (6, 6.5, and 7.4). The intracellular uptake of MR-SNC in MCF-7 cells and the DNA fragmentation were also studied.

## Materials and methods

### Materials

Sericin Bombyx mori (silkworm) powder (S5201, reagent grade, ≥ 99%) and all other chemicals, including *trans*-Resveratrol—3,4′—(CAS Number: 501-36-0 99%, HPLC, Fig. 1A), Melatonin (reagent grade, ≥ 98%, power, Fig. 1B), and nonsolvent acetone (ACS reagent, ≥ 99.5%) were acquired from Sigma Aldrich (St. Louis, USA). Ultrapure water (UP-Water) was produced using Milli-Q (Millipore, Bedford, MA, USA).

### Preparation of protein-based nanocapsules

To find the best protein concentration for preparing protein-based nanocapsules as a P-NDDS with a particle size of ≤ 200 nm, silk sericin (SER) was directly dispersed in UP-Water at various concentrations (0.1%, 0.2%, 0.3%, 0.5%, and 1% (w/v)) under moderate stirring at room temperature. Then, a protein-based nanocapsule was prepared via a flash-nanoprecipitation system adapted from Nelemans et al.^35^ with few modifications. Briefly, silk sericin solutions (0.1, 0.2, 0.3, 0.5, and 1% w/v) were added drop-wise (10 µl/5 s) into an organic phase of a non-solvent (acetone) under forceful stirring (800 rpm) at room temperature (Fig. S1). At that time, sericin-based nanocapsules were attained by completely evaporating the water and acetone to yield SER 0.1%—SER 1.

### Drug loading to sericin-based nanocapsules

The Resveratrol (RES) and Melatonin (MEL) solved in absolute dimethyl sulfoxide (DMSO) were loaded within sericin based nanocapsules via the direct system of dissolution into the aqueous phase (protein phase) at the final concentration of 0.6 mg/mL. The next step involved the addition of the dissolved RES + MEL aqueous phase into the acetone organic phase under forceful stirring (800 rpm) and pH 7.45 (3 wt% RES + MEL in blank-sericin based nanocapsules). This addition was done without the need for any tools or devices and only with an insulin syringe, 1 drop per 5 s, under intense stirring. Sericin-based nanocapsules (SNC) containing the drug were also recovered by completely evaporating water and acetone. Formerly, Intracellular Co-delivery of Resveratrol-melatonin utilizing a pH-responsive Sericin-based nanocarriers for anticancer therapeutics the antioxidants were loaded in optimal sericin-based-nanocapsule prepared formulations (0.1% w/v). The SNC was prepared in triplicate, freeze-dried, stored at − 20 °C, and protected from light. In addition to physical appearance, ease of reconstitution, and storage stability, the MR-SNC (Melatonin and Resveratrol-loaded sericin-based nanocapsule) was tested for variations in quality features, when added to cell culture media.

### Basic physicochemical properties of SNC and MR + SNC

Particle size, poly dispersity index (PDI), and particle surface charge (Zeta Potential) of SNC and MR-SNC were determined by Dynamic Light Scattering technique via Zetasizer instrument (Nano-ZS, Malvern Instruments Ltd., Worcestershire, UK) at 25 °C. At that time, other main characteristics of SNC and MR-SNC, including aggregation, size, morphology and shape were assessed with Scanning Electron Microscope (SEM) (EM3200, KYKY technology Co., China), at an operating voltage of 25 kV.

### Fourier transform infrared spectroscopy (FT-IR)

FT-IR spectroscopy was used to determine the chemical composition, molecular properties, and surface adsorption of nano-particles functional groups. Briefly, 1–2 mg of the pure antioxidants (RES/MEL), lyophilized blank SNC (B-SNC), and MR-SNC were mixed and then triturated with 100 mg potassium bromide (spectroscopy grade, Sigma Aldrich). The mixture was then placed in the FT-IR sample holder and pressed. Spectra were recorded over the scanning range of 200–4000 cm^-1^ with a spectral resolution of 4 cm^-1^ via FT-IR spectrophotometer (IR prestige-21, Shimadzu Co., Japan).

### Conformational analysis by circular dichroism (CD)

The structures of sericin, B-SNC, MEL, RES, MEL + RES, and MR-SNC dispersal were evaluated by circular dichroism spectrophotometer (J-1500, Jasco Co., Japan) via a 1 mm path length-quartz cell. Through this examination, the samples were scanned three times with a scan rate of 100 nm/min using ultraviolet light within 180–250 nm.

### Determination of entrapment efficiency (EE%) and drug loading (DL%)

Entrapment efficiency and drug loading were assessed by ultracentrifugation method^36^. At first, MR-SNC solution was ultracentrifuged (Optimal L-90 k, Beckman coulter Co, USA) for 15 min at 18,200 rpm to separate the free drug from MR-SNCs. Then 1 mL of supernatant was assessed via UV–vis spectrophotometry (Mini 1240, Shimadzu Co., Japan) for evaluating of the unentrapped RES or MEL at 297 and 277 nm, respectively^37^. Next, a standard calibration curve was prepared using various concentraions of RES and MEL (0.1–50 µg/mL for RES and 17.3–600 µg/mL for MEL, straight line with r^2^ = 0.98). The free drugs amounts were obtained using these curves. Finally, the entrapment efficiency (EE) and drug loading (DL) of RES and MEL on MR-SNC were calculated using the following formula:

$$\text{EE }(\mathrm{\%}) =\frac{\text{Total amount of drug added}-\text{Free amount of drug}}{\text{Total amount of drug added}}\times 100$$

$$\text{DL }(\mathrm{\%}) = \frac{\text{Total amount of drug added}-\text{Free amount of drug }}{\text{Total weight of nanoparticles}}\times 100$$

All the experiments were performed in triplicate, results are presented as mean ± SD.

### In vitro drug release

In vitro profiles of MEL and RES released from MR-SNC were studied at different pH values using a dialysis bag (Sigma Aldrich, 20 kD molecular weight cut-off) as described elsewhere^37–39^*.* Briefly, 5 mL of MR-SNC was placed in a dialysis bag, followed by immersion in a fixed volume of release medium (80 mL PBS, pH 7.45, 6.5, 6, adjusted by 1N HCl). Then, the bottels were incubated in an orbital mixer (Benchmark Scientific) for 55 h at 37 ± 0.5 °C at 300 rpm. At planned time intervals (0.5, 1, 2, 4, 24, 30, 48, and 55 h), 1 mL of PBS dialysate was removed and replaced with the same volume of fresh media and then analyzed by UV–vis spectrophotometry (Mini 1240, Shimadzu Co., Japan), at two different maximum absorption wavelengths (λmax), corresponding to 297 nm for RES and 277 nm for MEL. At each pH, the amount of MEL and RES released from MR-SNC was calculated as the drug release percentage at planned time intervals relating to the quantity of the entrapped drug.

### In vitro biological evaluation of MR-SNC at different pHs

#### Cell culture model

In the present study, the human breast adenocarcinoma cell line (MCF-7; NCBI No. C135^®^ Pasteur institute of Iran, Karaj, Iran) was chosen as an in vitro model because it preserves several features of differentiated mammary epithelium including the expression of functional estrogen receptor, responsiveness to estradiol and the ability to invade and metastasize. Furthermore, MCF-7 is a HER2-negative, and progesterone recepto- positive cell line^40,41^.

MCF-7 cells were cultured in Dulbecco’s modified Eagle medium (DMEM) supplemented with 10% fetal bovine serum (FBS) and 1% antibiotic–antimycotic solution (100 µg/mL streptomycin, 100 U/mL penicillin, and 0.25 µg amphotericin B) and was incubated at 5% CO_2_, 37 °C and humidified air atmosphere. Culture medium was renewed daily.

#### Cell viability assay

The growth inhibitory effect of RES + MEL (1, 5, 10, 20, 50, 100 and 200 µg/mL), B-SNC (5, 25, 50, 100, 250, 500 and 1000 µg/mL) and MR-SNC (5, 25, 50, 100, 250, 500 and 1000 µg/mL) at different pHs (7.4, 6.5, and 6) on MCF-7 cells was measured using a colorimetric MTT assay kit (Sigma Aldrich) as decribed previously^42,43^. Briefly, cells were plated in 96-well plates at a density of 3 × 10^3^ cells/well and, following overnight incubation, were treated with RES + MEL, B-SNC, and MR-SNC at the concentrations indicated above and using the media at different pHs for 24 and 48 h (Fig. S2). After the indicated times, 10 μL of MTT dye (3-[4,5-dimethylthiazol-2-yl]-2,5-diphenyl tetrazolium bromide, 5 mg/mL in phosphate buffered saline), was added to each well, and the plates were incubated for 4 h at 37 °C. Then, the media was aspirated, and 100 μL of the solubilization solution (10% SDS in 0.01 M HCl) was added to each well to solubilize the formant crystals. The absorbance of the plates was measured on a spectrophotometric plate reader (ELx808, Lonza BioTek Co, Switzerland), at a wavelength of 570 nm. The percentage of viable cells was calculated by following formula, and half-maximal inhibitory concentration (IC50) was obtained.

$$\text{Cell viability }(\mathrm{\%}) =\frac{A 570 \left(Sample\right)}{A 570 \left(Control\right)}\times 100.$$

#### Interacellular uptake and DAPI staining

To study the cellular uptake of the fabricated nanoparticle, the cells were stained with FITC as described^41^. First, a suspension of MR-SNC with a concentration of 1 mg/mL was mixed with the same volume of FITC (5 µg/mL) and stirred for 10 h in the dark at room temperature. Then the suspension was centrifuged at 13,000 rpm for 10 min and washed with PBS three times. Next, MCF-7 cells were seeded at a density of 2 × 10^5^ cells per well in 6-well culture plates. After discarding the medium, the cells were washed twice with PBS. Next, cells were treated with 1000 µg/mL of stained MR-SNC at pH 6 (obtained from the MTT assay) for 8 h. Then the cells were washed three times with PBS, and 4% paraformaldehyde (100 µL/well) was added to fix the MCF-7 monolayers for 15 min.

Nuclear evaluation was also studied by DAPI (4',6-diamidino-2-phenylindole; 40 µM) staining. After 24 h incubation, the cells exposed to the MR-SCN at IC50 concentrations were washed with PBS, fixed with 4% (v/v) paraformaldehyde for 30 min, and rinsed twice with PBS. Next, DAPI was added for 20 min in the dark to stain the nucleus. Finally, cells were rinsed with PBS 3 times, and the well (n = 3) was evaluated by cytation cell imaging reader (Cytation 5, Biotek Co., USA).

### Statistical analysis

The obtained data were analyzed using SPSS for Windows, version 23.0, and GraphPad Prism 8 software. The one-way analysis of variance (ANOVA) was used to determine the statistically significant differences between the groups. All the experiments were performed in triplicate, and mean values ± standard deviation (SD) were used to present the data. A p-value less than 0.05 was considered to be statistically significant.

### Ethics approval and consent to participate

The current study was approved by the Ethics Committee of the Kermanshah university of medical science (IR.KUMS.REC.1400.331).

## Results

### Development and characterization of protein-based nanocapsules

**Figure 2:**
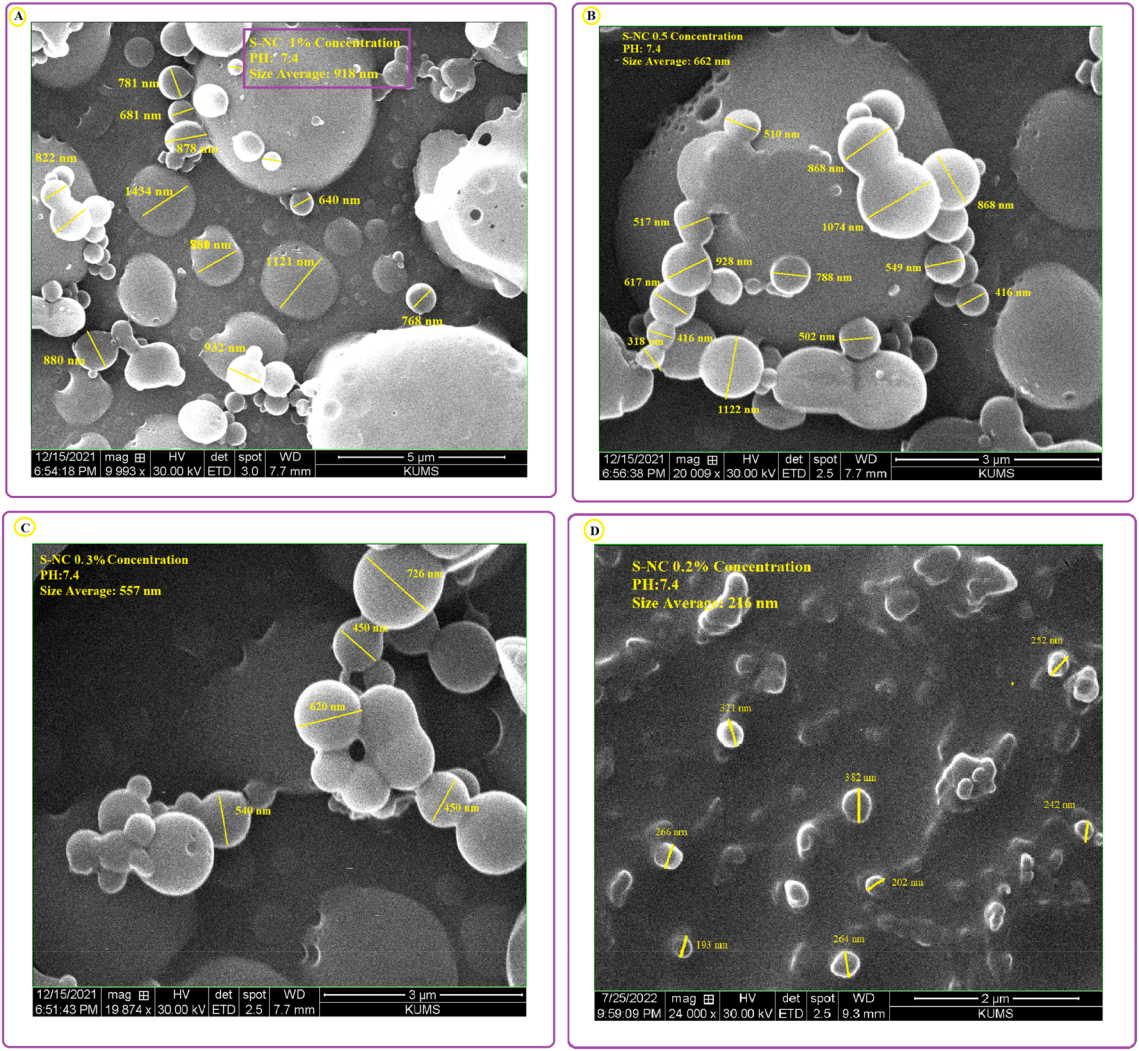
SEM analysis of (**A**) 0.2% sericin nano-capsule with the average size of 265 nm; (**B**) 0.3% sericin nano-capsule with the average size of 557 nm; (**C**) 0.5% sericin nano-capsule with the average size of 662 nm, and (**D**) 1% sericin nano-capsule with the average size of 918 nm.

**Figure 3:**
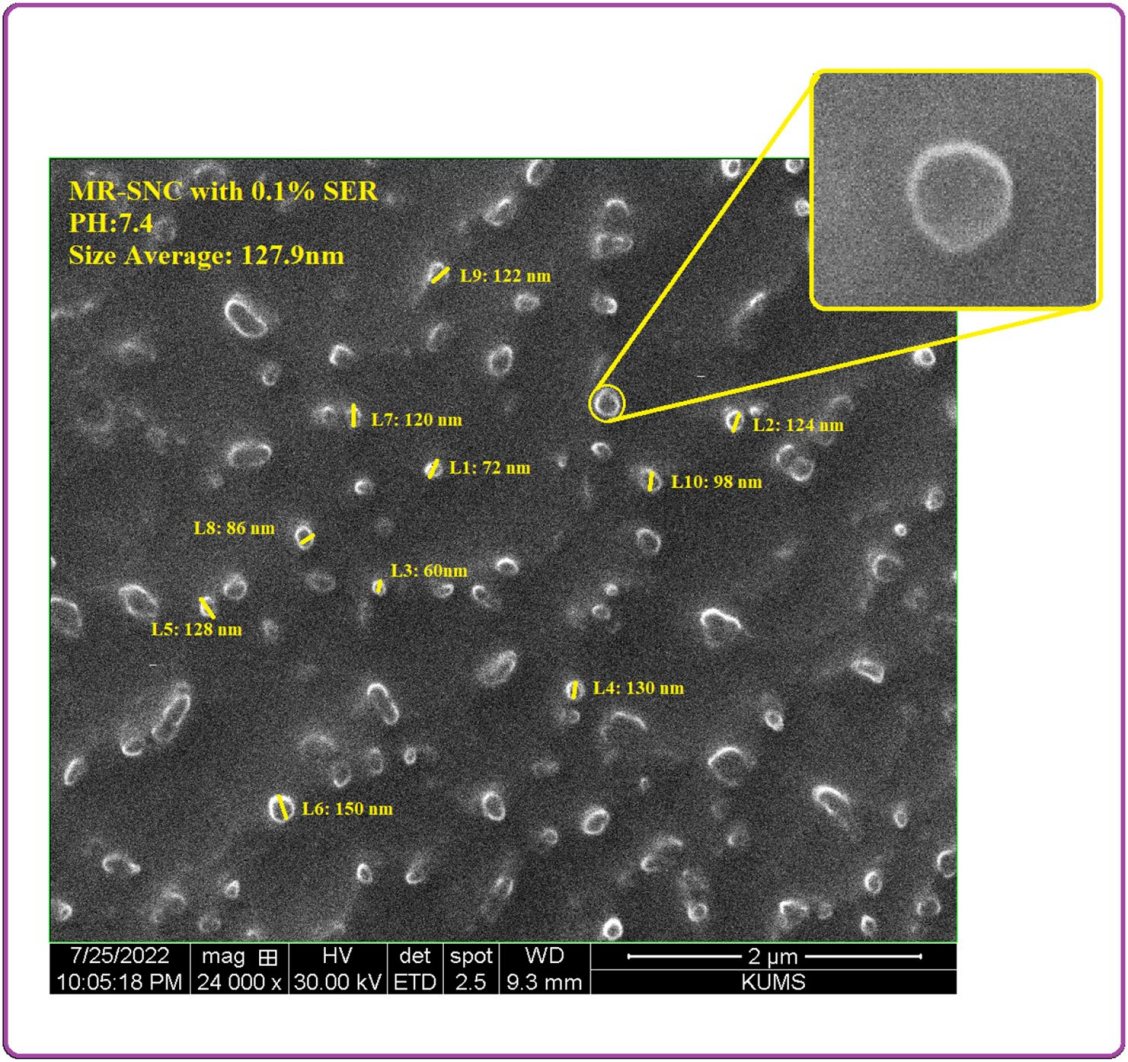
SEM analysis of 0.1% sericin nano-capsule with the average size of 127.9 nm.

**Figure 4:**
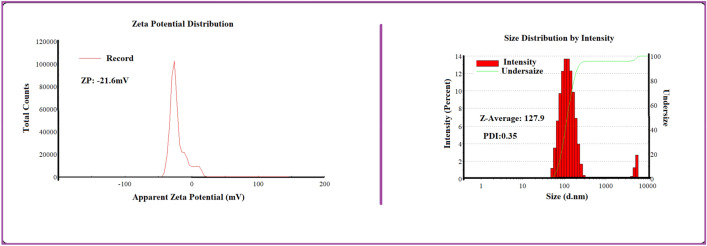
Physicochemical characterization of NDCDS (RES + MEL-SCN).

Blank SNCs were prepared through the process of nanoprecipitation^35^. Physico-chemical characteristics of all sericin nanoparticles were evaluated in different sericin concentrations (Fig. 2). The results showed that the MR-SNCs made of 0.1% sericin, were regularly distributed and had a circle shape with an average diameter of about 109 nm (Fig. 3). Based on these studies, the 0.1% sericin formulation was used throughout the experiments. DLS measurements of MR-SNC determined an average size of 127.9 nm and a size distribution (PDI) of 0.356 (Fig. 4, Table 1). The zeta potential of these nano-capsules was -21.6 mV, (Fig. 4, Table 1). The zeta potential value determines the nanoparticle's stability and cellular uptake^44^. In contrast, SEM specifies the dry particle size. The average diameter obtained by SEM was lower than the size measured by Zetasizer. Considering that, the zetasizer measures the diameter of the particle in the solution and the particles hydration increases their size. The amount of EE (%) in a single MR-SNC was 98%. The antioxidants were loaded in the SNC, with a DL (%) of approximately 27 (Table 1).

**Table 1:** Physico-chemical features of MR-SNC.

	Z-average diameter (nm)	Polydispersity index	Zeta potential (mV)	DL (%)	EE (%)
MR-SNC	127.9	0.356	− 21.6	27	98

### FT-IR analysis

**Figure 5:**
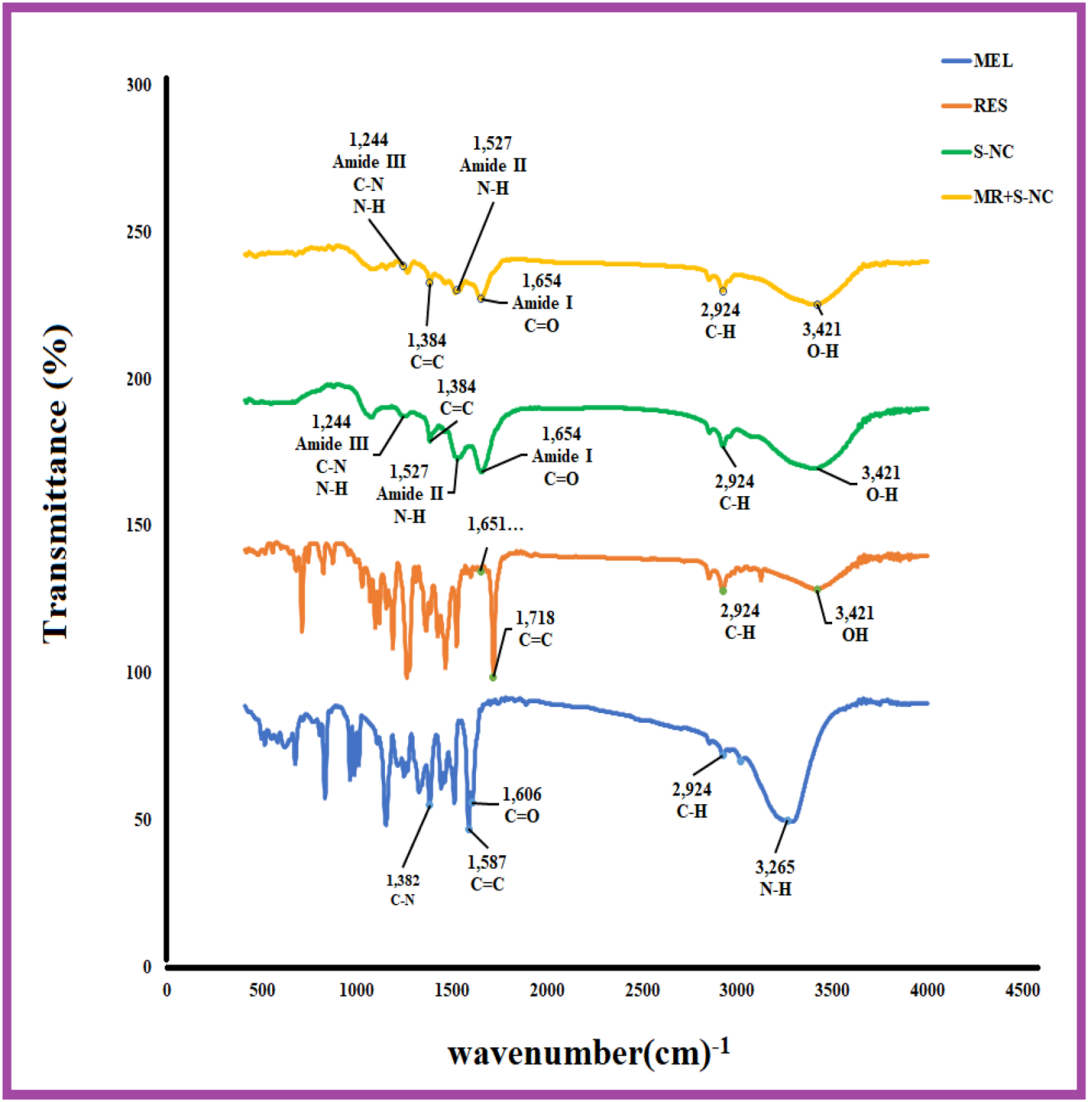
The FT-IR spectra of melatonin (MEL), Resveratrol (RES), SNC, and MR-SNC.

Figure 5 shows the FT-IR spectra of MEL, RES, SNC, and MR + SNC. The spectrum of MEL (Figs. 5, S3a), revealed sharp bands at 3265, 3018, 2924, 2852, 1606, 1587, 1512, 1382, 1327, 1265, 1247, 1151, 1010, 987, 964, 833, and 675 cm^−1^ and the broadband at 1600–2500 cm^−1^. The peak at 3265 cm^−1^ corresponds to N–H stretching vibration. The 3018 cm^−1^ peaks revealed CH vibration. The broad peak at 2924 cm^−1^ was the feature of hydroxyl groups (OH) stretching vibration. The band at 1606 cm^−1^ is related to C = O stretching vibration, and the peaks at 1587 and 1382 cm^−1^ correspond to the C = C and C-N stretching, respectively. The RES spectrum (Figs. 5, S3b) showed peaks at 3421, 3124, 3018, 2922, 1718, 1651,1598, 1558, 1523, 1465, 1425, 1386, 1365, 1340, 1276, 1261, 1188, 1155, 1095, 1070, 1029, 993, and 711 cm^−1^. The broad peaks at 3124–3421 cm^−1^ are a feature of OH stretching vibration. The sharp peak at the 1651 cm^-1^ region is related to the double bands C = C the carboxylic acid carbonyl stretching vibration. The C–C stretching vibrations of RES were observed between 1465 and 1558 cm^-1^.

Since the Blank-SNC is comprised of Sericin (S) alone; and after the freeze-dry proceedure, the FTIR peaks for both SNC and sericin powder (S) were quite similar, only one of them is displayed in the FTIR diagram (Figs. 5, S3c). The FT-IR spectrum of the prepared SNC designated sharp bands at 3400, 3082, 2924, 2852, 1654, 1527, 1462, 1384, 1244, 1159, 1076, and 877 cm^−1^. In this FTIR spectra, individual amide absorption bands were detected at 1654 cm^−1^ (amide I, C=O stretching), 1527 cm^−1^ (amide II, N–H bending), and 1244 cm^−1^ (amide III, C–N stretching, and N- H bending), which suggest the presence of a random coil structure. Another band at 1384 cm^−1^ was credited to C–H and O–H stretching and C–OH banding of hydroxyl amino acid side chains, like serine.

MR-SNC was synthesized by three monomers; MEL, RES, and Sericin. In the spectrum of MR-SNC, all the bands relating to MEL, RES, and SNC were identified. As shown in Figs. 5 and S3d, the sharp bands at 3402, 2924, 2852, 1653, 1587, 1537, 1516, 1465, 1384, 1323, 1267, 1188, 1151, 1074, 968, 875, 713, and 677 cm^−1^ were observed in MR-SNC FTIR spectrum. In this spectrum prominent shifting of the peak was observed from 1527 to 1537, 1462 to 1465, 1244 to 1267, 1159 to 1188, and 877 to 875 cm^-1^ in comparison to those of SNC. Additionally, comparative peaks with SNC in this spectrum were of higher intensity as opposed to the SNC, which was due to the formation of intermolecular hydrogen-bonding between NH and OH group of SNC with MEL and RES. Moreover, the new peaks appeared at 1587, 1516, 1323, 1188, 968, 713, and 677 cm^−1^ at the MR-SNC FTIR spectrum, indicating a shoulder peak in the spectra of MR-SNC, signifying MEL and RES were joined into SNC. Simultaneously, the expanded intensity of amide I, II, and III demonstrated the construction of a new amide linkage in MR-SNC. As opposed to SNC, a shoulder peak at 677–1500 cm^−1^ was seen in the spectra of MR-SNC, which could be to the C=O vibration of the C–H bunch from MEL and RES. These results confirmed the presence of MEL and RES in the spectrum of MR-SNC.

### Stability of sericin secondary structures

**Figure 6:**
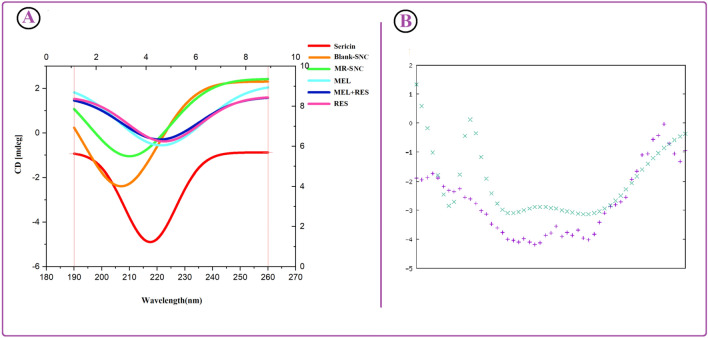
CD spectra of sericin, B-SNC, MR-SNC, MEL, MEL + RES and RES (**A**); sericin spectrum demonstrated a weak negative band at 218 nm, which was assigned to β-sheet. The K2D3 software output of sericin secondary structure (**B**).

Circular dichroism (CD) is a sensitive technique to monitor the conformational changes in a protein. CD, as a biophysical method, was used to probe sericin structure following its formation of nanocapsules by the nanoprecipitation method. It measures a difference in the absorption of right and left circularly polarized light to determine a protein’s secondary structure. Sericin has a molecular weight of 198.6 kDa with 1758 amino acid residues. As its spectra reveal (Figs. 6A, S4), sericin showed a weak negative band at 218 nm, which was assigned to the β-sheet. Figure 6 confirms that the CD spectra of blank-SNC showed a good similarity to the native sericin spectrum, a weak negative band at 208 nm. The CD spectra of MEL, RES and MEL + RES were also obtained. These compounds showed the similar broad negative bands between 200 and 240 nm. The CD spectra of MR-SNC confirm that the binding of RES/MEL to sericin causes only a decrease in negative ellipticity at all wavelengths of the far-UV CD without any significant shift of the peaks, which clearly indicates the lack of changes in the protein secondary structure, and a less decrease of the α-helix content in protein (Fig. 6). The protein secondary structure from CD spectra was also estimated by K2D3 software (Fig. 6B). It demonstrated that there was 10.27% β-sheet and 4.6% α-helix in intact sericin (Fig. 6B). Therefore, the result suggests nanoprecipitation successfully preserves the primary and the secondary structures of the protein. Also the occurrence of a small conformational change at the secondary structural level in the reaction between RES/MEL and Sericin in MR-SNC was detected.

### In vitro drug release

**Figure 7:**
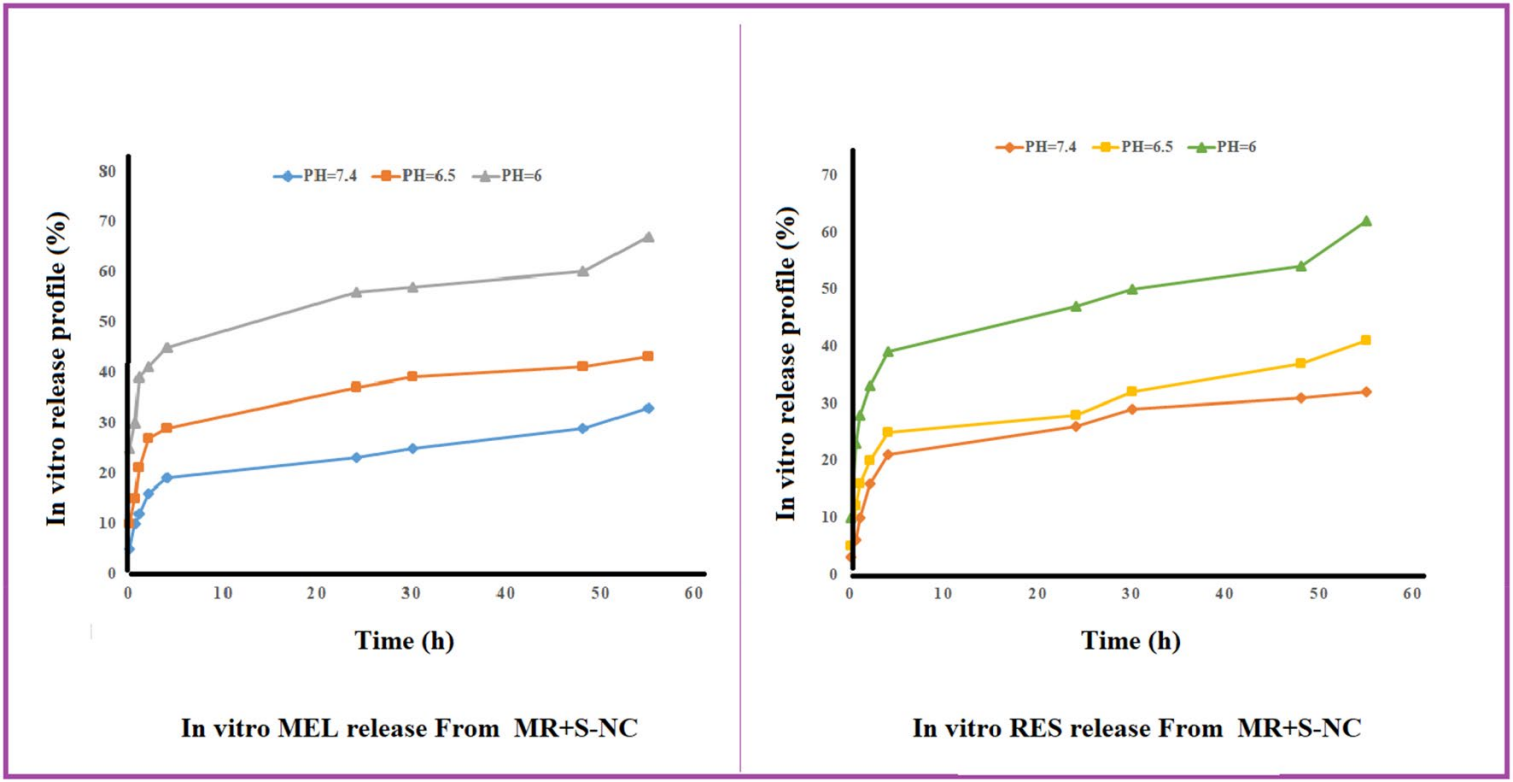
The in vitro release of MEL and RES from MR-SNC under different pH values (7.4, 6.5, 6), up to 55 h.

The in vitro release of MEL and RES from MR-SNC was examained by the dialysis bag method, under different pH values (7.4, 6.5, and 6), up to 55 h (Fig. 7). Based on our results, at 24 h, 26, 28, and 47% release was observed for RES at pH of 7.4, 6.5 and 6, respectively. At 55 h, 32 and 41% of RES were released at pH of 7.4, and 6.5 respectively; with an apparent burst release (62%) at pH 6.

In comparison, MEL being released from MR-SNC was 23, 37 and 56% at 24 h in pH of 7.4, 6.5 and 6, respectively. At 55 h, 33 and 43% of MEL was released from the corresponding nano-capsule at pH of 7.4 and 6.5, respectively. On the other hand, an apparent burst release (67%) of MEL occurred in pH 6 at 55 h.

### In vitro efficacy evaluation

#### Cell Viability

To assess viability of the MCF-7 cells after treatment with RES + MEL (1, 5, 10, 20, 50, 100 and 200 µg/mL), B-SNC (5, 25, 50, 100, 250, 500 and 1000 µg/mL) and MR-SNC (5, 25, 50, 100, 250, 500 and 1000 µg/mL) at different pHs (7.4, 6.5, and 6), the MTT assay was done. There were no significant differences between controls and treated groups after 24 h (data not shown).

**Figure 8:**
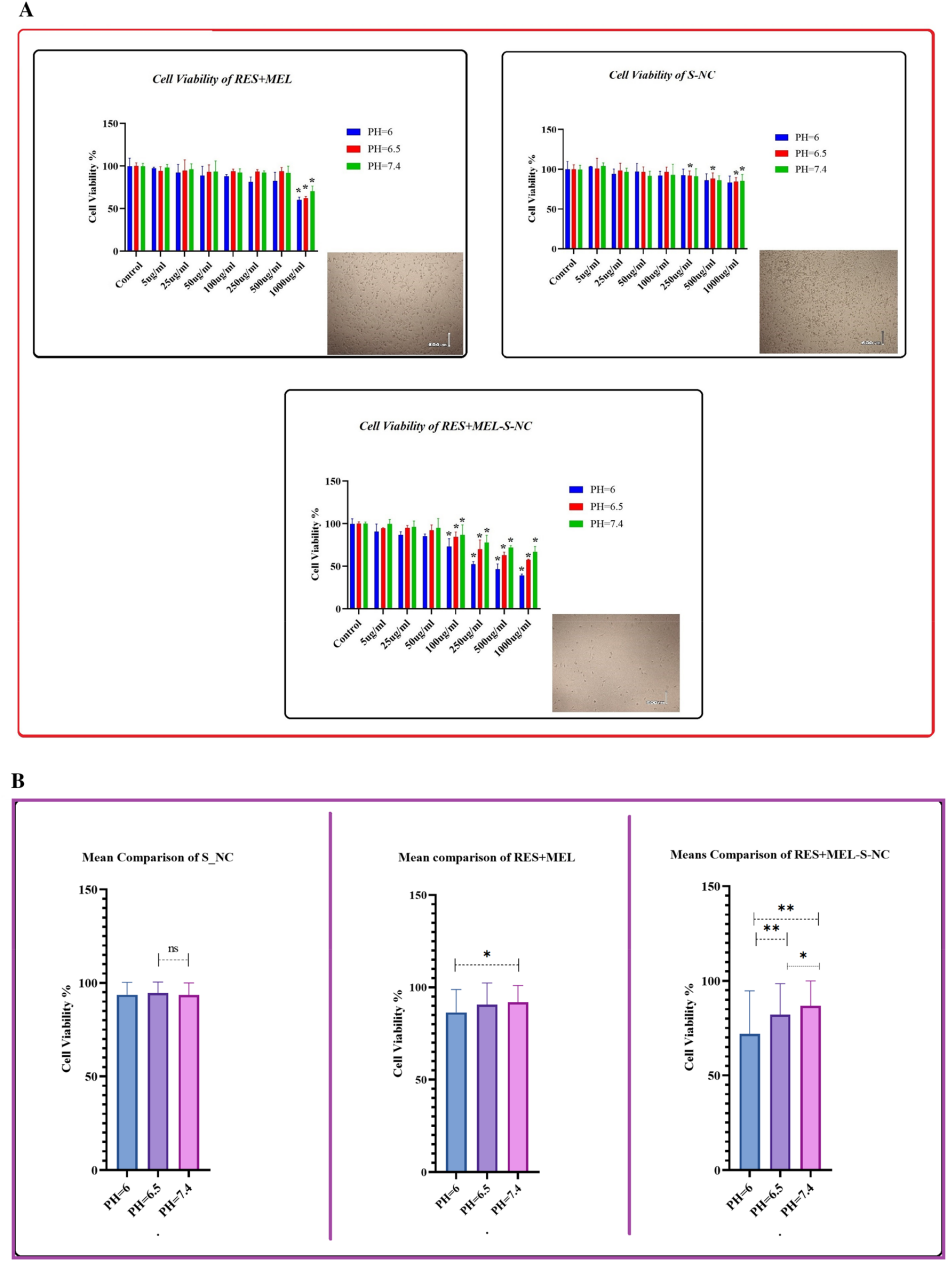
(**A**) MCF-7 cells viability after treatment with various concentrations of B-SNC, RES + MEL, and MR-SNC at different pHs. Results are mean ± SD for 3 different experiments, *P ≤ 0.05 compared to the respective controls. (**B**) Means Comparission of MCF-7 cells viability at various pHs in three groups. Results are mean ± SD for 3 different experiments, *P ≤ 0.05 compared to the respective controls.

Exposure to RES + MEL (1–100 µg/mL) for 48 h also showed similar cell viability to the control group. However, at 200 µg/mL, the cell viability significantly decreased at all pH values (7.4, 6.5 and 6) (Fig. 8A). Generally, pH 6 was more effective than the physiologic pH (7.4) in this group (Fig. 8A). On the other hand, 48 h treatment with 250, 500 and 1000 µg/mL of B-SNC, significantly reduced cell viability at pH 6.5 in comparison to the control group (Fig. 8B). Comparison of the mean values indicated that different pH values had similar effects on treatment with various concentrations of B-SNC (Fig. 8B).

Treatment with MR-SNC for 48 h caused significant cell death at all concentrations and pH values. None of the other treatments induced cell viability alterations (Fig. 8A). The results also showed that the viability of MCF-7 cells at pH 6 was significantly lower than that at pH 6.5 and 7.4, and we just observed IC_50_ values of 500 μg/mL at pH 6 (Table 2). Also, at pH 6.5 MCF-7 cell viability was significantly different from that at pH 7.4 (Fig. 8B).

**Table 2:** MCF-7 cells viability (%) after treatment with various concentrations of MR-SNC at different pH values.

Concentration of MR-SNC (µg/ml)	Cell viability (%) at pH 6	Cell viability (%) at pH 6.5	Cell viability (%) at pH 7.4
5	91.8	94.6	99.7
25	87.1	95.2	96.3
50	85.4	92.4	95.0
100	73.2	84.5	86.9
250	52.3	69.9	78.0
**500**	**46.6**	**63.0**	**72.1**
**1000**	**39.4**	**57.5**	**66.8**

Significance values are in bold.

#### Intracellular uptake and DAPI staining

**Figure 9:**
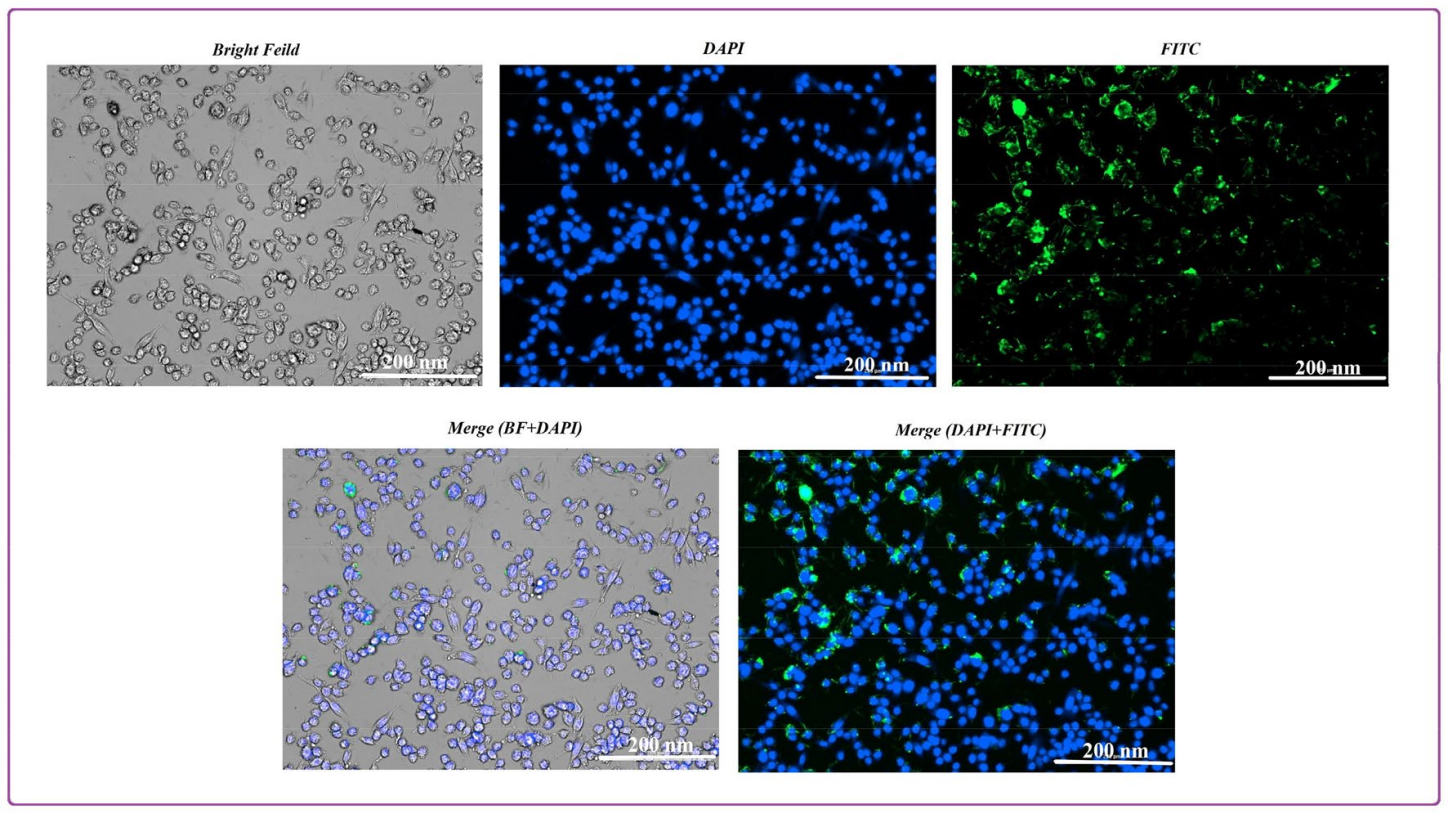
Intracellular uptake of FITC labeled MR-SNC (500 µg/mL, pH 6) and DAPI staining of MCF-7 cells. Bar scale, 200 nm.

A representative fluorescence microscopy image of the morphology of the MCF-7 cells treated with FITC-labeled MR-SNC at its IC_50_ (500 µg/mL) after 4 h exposure at pH 6 is shown in Fig. 9. The results show that the FITC-labeled-MR-SNC was taken up by the cells, potentially through endocytosis and was distributed in the cellular cytoplasm through interaction with the cytoskeleton fibers. Moreover, Fig. 9 shows the presence of cells stained by DAPI. After crossing the membrane, DAPI binds to DNA A-T rich regions and shows nuclear condensation and DNA fragmentation^45^. According to the captured images (Fig. 9), MR-SNC treated cells showed considerable degree of DNA fragmentation, chromatin condensation and apoptosis. Merged fluorescent DAPI and FITC images for MR-SNC are also shown in Fig. 9.

## Discussion

The utility of single or multiple antioxidant supplementations is a reasonable chemopreventive/chemotherapeutic strategy by scavenging free radicals and reducing oxidative stress. However, there are challenges in such an approach due to poor permeability, low solubility, in vivo instability, and poor bioavailability. The use of nanotechnology can overcome these problems and enhance efficiency through targeted drug delivery to the tumor site^46–48^.

Nanomedicine is a rapidly emerging field in cancer diagnosis and treatment. Protein-based nanocarriers as organic nano-vectors, have a remarkable potential to be used in oncology. These nanocarriers are stable, biodegradable, biocompatible, chemically modifiable, non-toxic, and non-immunogenic. The ease of fabrication and the abundance of its raw material (protein) in nature are its other advantages^49,50^. Regarding the role of protein nanocarriers in drug delivery, we aimed to utilize a sericin nanocarrier, to deliver the optimal dose of MEL and RES in the form of MR-SNC and to investigate their synergistic antioxidant-efficacy on MCF-7 breast cancer cell line.

**Figure 10:**
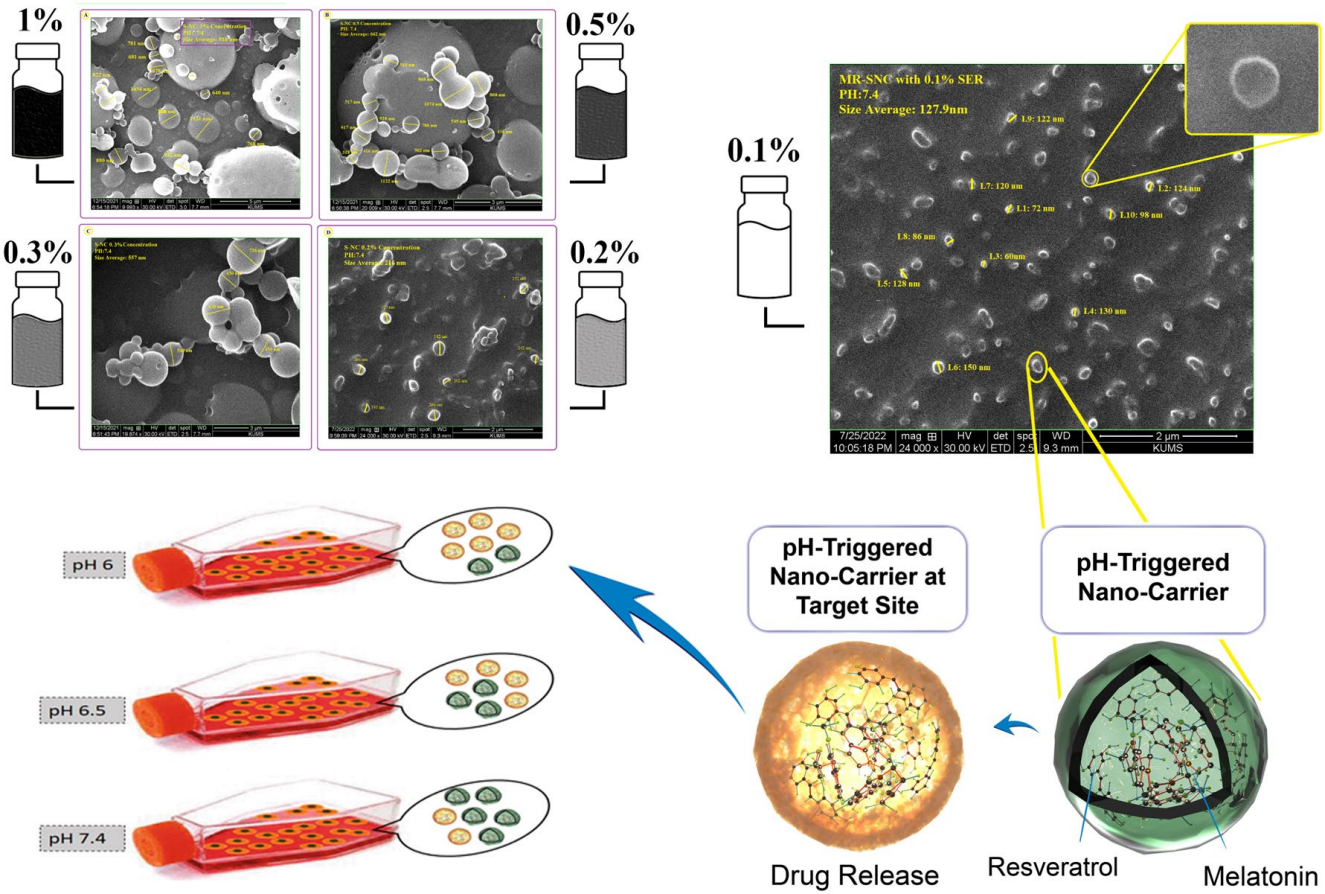
The charge reversal process may disrupt the electrostatic interactions between the sericin amino acids side chains (carboxyl and amino) in SNC at cancer environment PH, releasing MEL and RES.

Protein concentration is one of the most important factors determining the size of a protein based nanocarrier. As the protein concetration increases, the nanoparticle size distribution gets larger^51^. We tested five different sericin concentrations (0.1%, 0.2%, 0.3%, 0.5%, and 1% (w/v)) for protein-based nanocapsules preparation to find out the optimum concentration (Figs. 2, 10). The physicochemical analysis indicated that 0.1% (w/v) was the best concentration for this purpose. Hence we applied 0.1% sericin nanocapsule throughout the next experiments (Fig. 3). Our finding is in line with a previous study where the lowest concentration of sericin (0.1%), showed the smallest nanoparticle size and aggregation and the highest release efficiency and drug content^52^. In addition, smaller particles can release entrapped drugs faster compared to large particles due to their larger surface area^51^. Our result also domenstrated that drug release from the sericin-based nanocapsule was pH-dependent.

The tumor microenvironment is of utmost importance. Within solid tumors, pH values range from 6.0 to 7.0 due to carbonic anhydrase activity and lactic acid production through anaerobic glycolysis. This environment can make nanocarriers sensitive to acidic pH through the protonation of its functional groups and changing of the nanoparticle surface charge from negative/neutral to positive that makes the carrier collapse and release its carrying drug cargo^53^.

Sericin is a hydrophilic protein, that is composed of 40% serine and aspartic acid with a high potential for self-assembling. The carboxyl groups of aspartic acids render sericin a net negative charge at neutral pH. As can be seen in Fig. 10, thses ionizable groups can undergo protonation in mild acidic conditions of the cancer microenvironment^54,55^. This charge reversal process may disrupt the electrostatic interactions between the sericin amino acids side chains (carboxyl and amino) in SNC, releasing MEL and RES^24^. Furthermore, MEL and RES are more stable at low pH values^56,57^. This stability in the acidic environment can potentially help with SNC diffusion.

MEL and RES had a significant toxicity on MCF-7 cell viability at 48 h, especially at 200 µg/mL and all pH values. SNC also reduced cell viability at 250, 500 and 1000 µg/mL at pH 6.5. Sericin has a remarkable antioxidant activity via preventing lipidic peroxidation and oxidative stress^58^. At low doses it has antioxidant and protective effects on normal and tumor cells. However, the high doses it shows toxicity towards cancer cells^46^. Our results are consistent with these observations. Also, the cytotoxic effects of MR-SNC on the viability of the MCF-7 cells was observed at 100, 250, 500 and 1000 µg/mL at all pH values and was significantly higher than that exposed to SNC after 48 h. This finding strongly suggests potential synergy between the three antioxidants (sericin, MEL and RES).

Our result with MR-SNC after 48 h showed high cell toxicity at pH 6 accross the concentrations tested. Thses results are consistant with the charge-reversal property of MR-SNC at low pH values.

Our results with FITC showed high fluorescent signal. Cancer cells have a high negatively-charged surface due to high concentration of glycoproteins on their membrane. Many of these glycoproteins contain sialic acid, which in part contributes to the negative charge cell membrane^59^. In addition, only positively-charged nanoparicles can attach to cencer cells^60,61^. Our uptake studies at pH 6 altered the nanocarrier surface charge from negative to positive via increasing the amino/carboxyl ratio in MR-SNC through a charge reversal process^24^. So, FITC-labeled MR-SNC attached to the MCF-7 membrane quite easily and was picked up by these cells via transcytosis.

Our DAPI results illustrated chromatin condensation and DNA fragmentation in MCF-7 cells. DNA damage is an apoptosis hallmark, so our results suggest that the MEL and RES encapsulation in SNC leads to apoptosis of MCF-7 cells. Sericin based nanocarriers can induce cell apoptosis via caspase-3 activation, Bax proteins upregulation and Bcl-2 downregulation^55^. At the time of conducting this research, it was not possible for us to investigate intracellular and extracellular ROS, due to the deficit and high cost of the required materials and equipment, in our country due to the sanctions condition that it was a limitation for present work.

## Conclusions

In summary, we created a pH-sensitive nanocarrier with a low sericin concentration by nanoprecipitation as a simple and reproducible method. MEL and RES were loaded in this nanocarrier with charge-reversal property, that turned its surface charge from negative to positive in a mildly acidic environment. Our morphological and analytical investigations indicated the intact sericin, MEL, and RES structures after nanocarrier preparation and the desirable size, shape and size distribution of nanoparticle. The pH-dependent drug release behavior of MR-SNC, with the maximum release in pH 6, showed this smart strategy's potential efficacy in releasing antioxidants in the acidic tumor microenvironment. Moreover, MR-SNC significantly decreased MCF-7 cell viability in acidic pH. MR-SNC was picked up by MCF-7 cells in pH 6, accumulated inside them and caused DNA damage. In conclusion, the charge-reversal protein-based nanocarrier will be an excellent strategy to maximize the efficiency of anti-cancer agents and minimize the drug’s unspecific toxicity.

## Supplementary Information

Supplementary Figures.

## Data Availability

The datasets generated and/or analyzed during the current study are not publicly available due to privacy and ethical issues, but are available from the corresponding author on reasonable request.
