# Supplementary material for: Codelivery of resveratrol melatonin utilizing pH responsive sericin based nanocarriers inhibits the proliferation of breast cancer cell line at the different pH
Source: Sci Rep. 2023 Jul 8;13:11090. doi: 10.1038/s41598-023-37668-y (PMC10329705; doi:10.1038/s41598-023-37668-y)
Supplement: Supplementary file 1 — Supplementary Figures. [file 41598_2023_37668_MOESM1_ESM.docx]

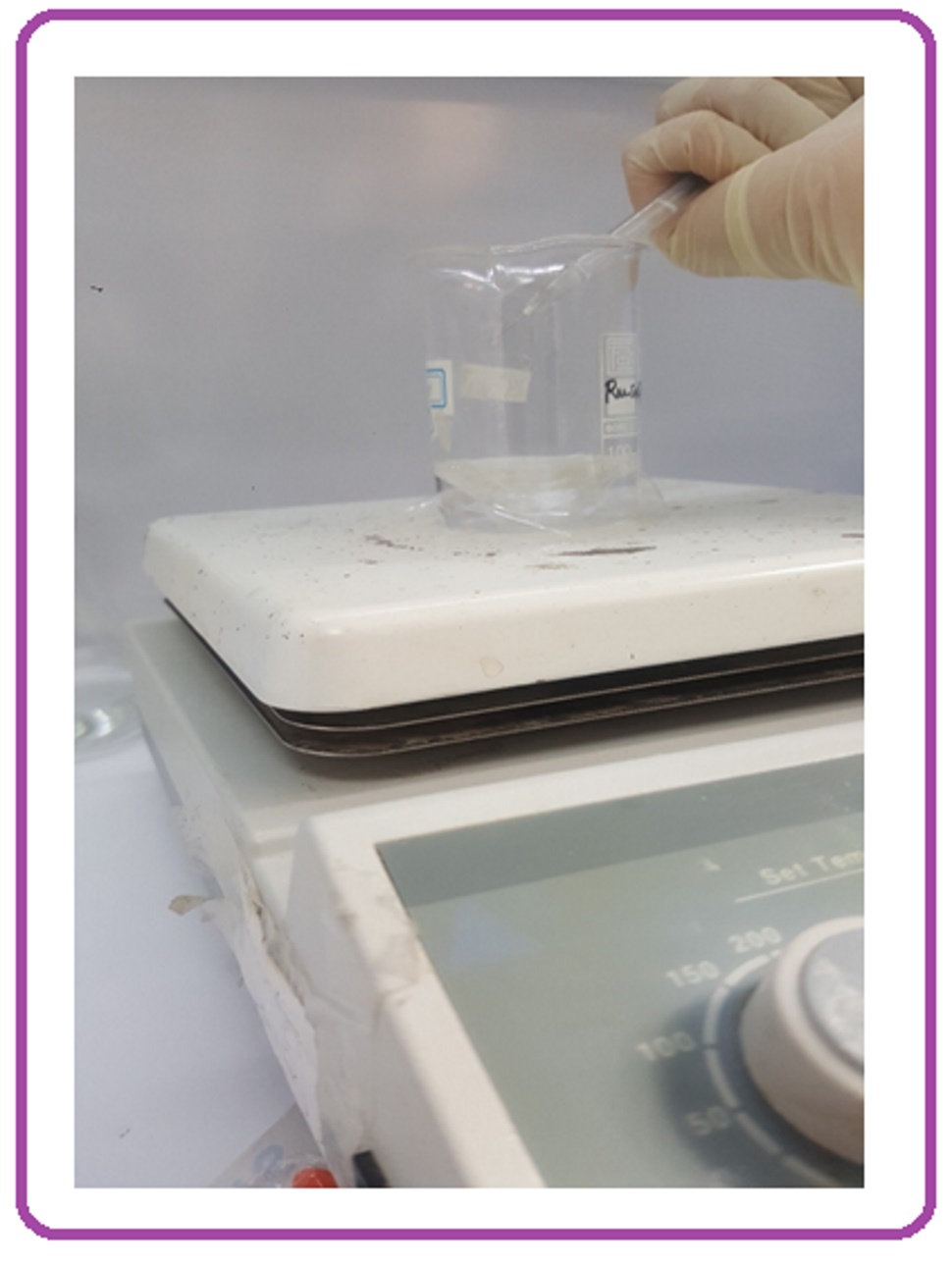


Fig. S1. The sericin-based nanocapsules preparation with an insulin syringe and drop-by-drop flow under intense stirring


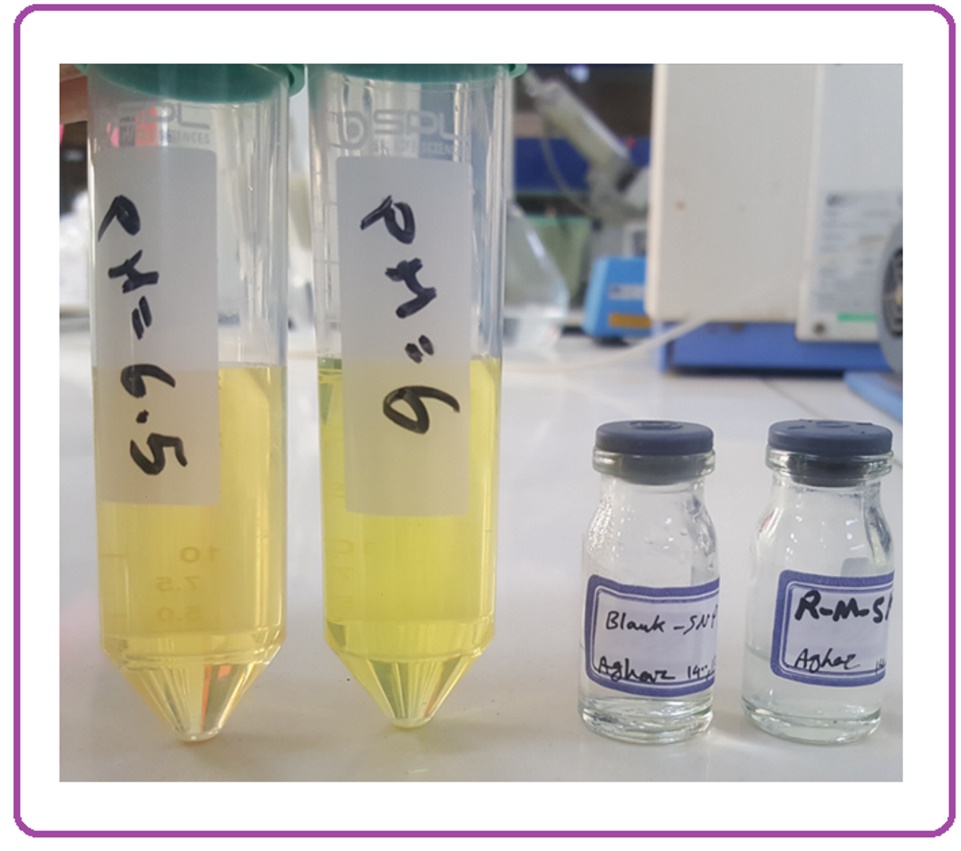


Fig. S2. The culture media with different pH values were used for assessing the cellular toxicity of B-SNC, MEL+RES and MR-SNC. (Picture does not show the medium with pH 7.4 and MEL+RES.)


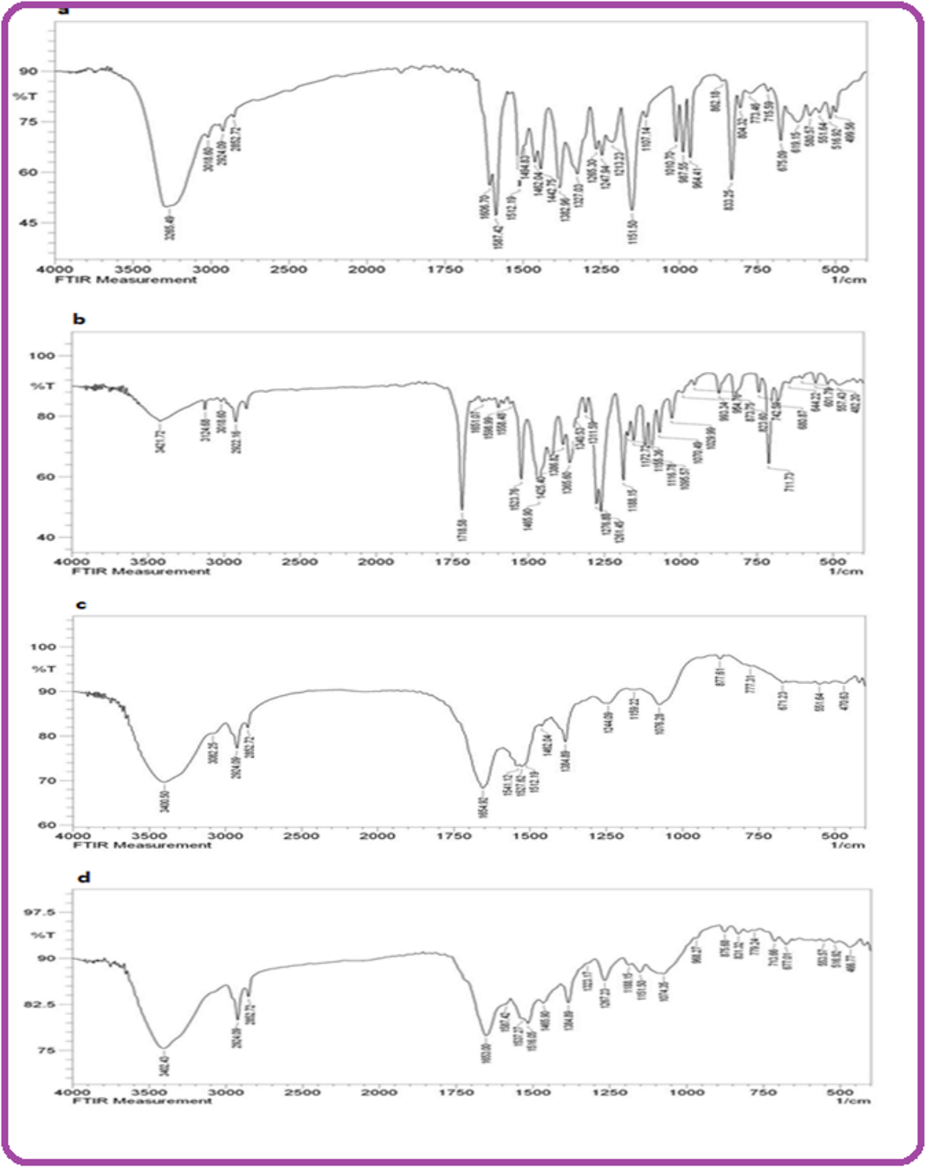


Fig S3. The FT-IR spectra of a) melatonin (MEL), b) Resveratrol (RES), c) SNC, and d) MR+SNC.


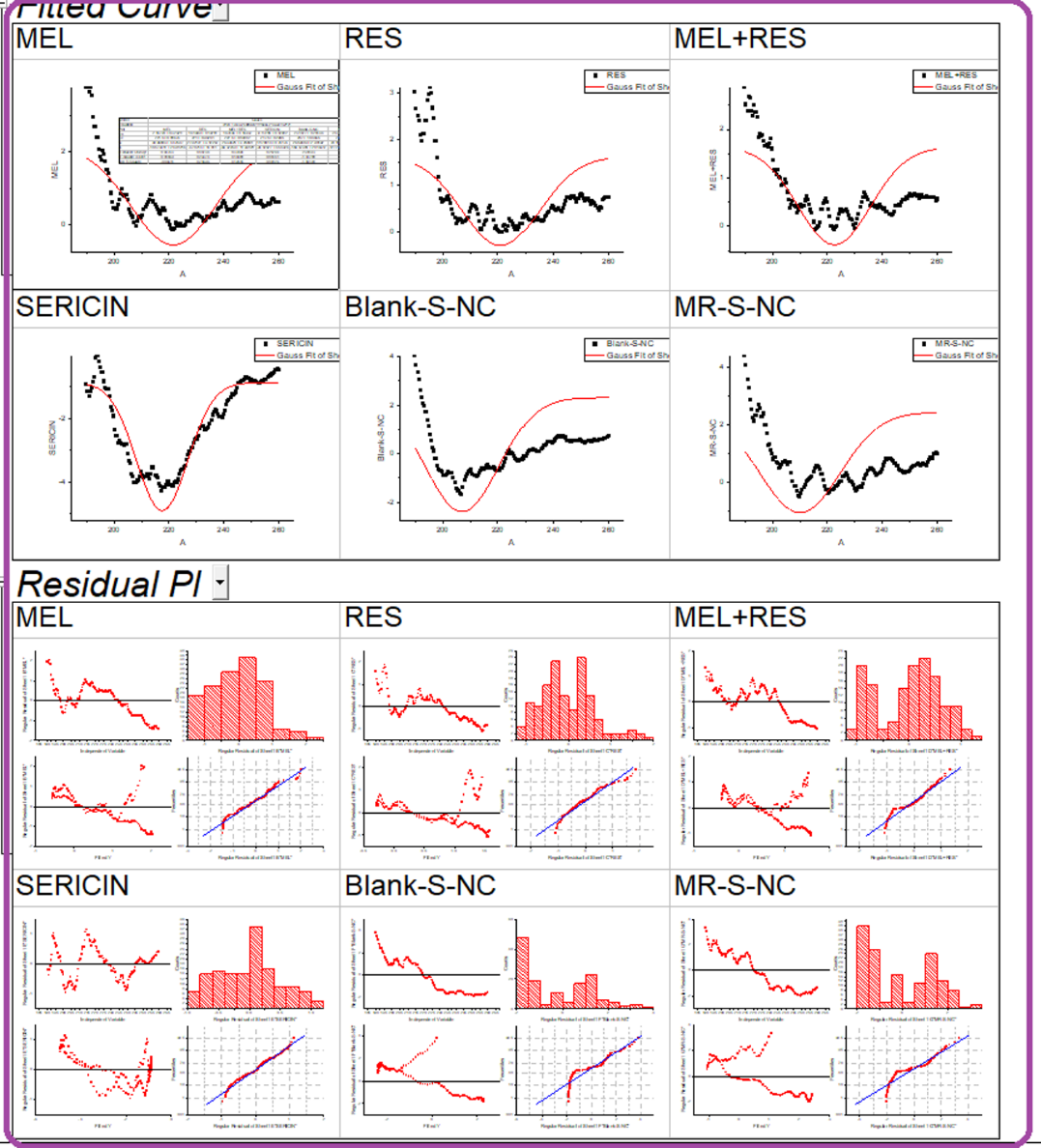


Fig S4. CD spectra of sericin, B-SNC, MR-SNC, MEL, MEL+RES and RES. Sericin and B-SNC spectra showed a notable similarity, declared that the preparation method had no impact on sericin structure.
